# Expression of Recombinant Rat Secretable FNDC5 in Pichia Pastoris and Detection of Its Biological Activity

**DOI:** 10.3389/fendo.2022.852015

**Published:** 2022-03-07

**Authors:** Yi Zhao, Hui Li, William Donelan, Shiwu Li, Dongqi Tang

**Affiliations:** ^1^ Center for Gene and Immunotherapy, The Second Hospital, Cheeloo College of Medicine, Shandong University, Jinan, China; ^2^ Department of Urology, College of Medicine, University of Florida, Gainesville, FL, United States; ^3^ Department of Pathology, Immunology and Laboratory Medicine, College of Medicine, University of Florida, Gainesville, FL, United States

**Keywords:** FNDC5, irisin, browning, lipolysis, obesity

## Abstract

FNDC5 is the precursor of the myokine irisin proposed to exhibit favorable metabolic activity, including anti-obesity and anti-diabetes effects. The diversity of FNDC5 transcripts has been reported by several studies, but the role and existence of these transcripts are not well defined. In our previous study, a novel secretable FNDC5 (sFNDC5) isoform lacking the transmembrane region was found in rat INS-1 cells and multiple rat tissues. In the current study, we established a high-yield system for the expression and purification of sFNDC5 in *Pichia pastoris*, and functional investigations were undertaken using 3T3-L1 cells. We discovered that this new isoform has similar and even stronger biological functions than irisin, which may be due to its more complete structure without cleavage. Hence, we believe that sFNDC5, as the first identified readily secretable derivative, can better induce lipolysis and can potentially prevent obesity and related metabolic diseases.

## Introduction

Obesity, which is associated with the development of various metabolic diseases, has been highlighted as a priority public health problem worldwide in recent decades (1, 2). Excess weight puts people at higher risk for chronic conditions such as diabetes mellitus, hypertension, insulin resistance, cardiovascular diseases, and even cancers (3). Since an increase in the number and/or size of adipocytes is the main characteristic of obesity, it is thought that the key to overcoming obesity is to increase lipid metabolism. Hence, a focus on the study of adipocytes is regarded as the primary means to solve the long-term dysregulation of energy balance (4).

White adipose tissue (WAT) and brown adipose tissue (BAT) are two typical types of adipose tissues with opposite functions. The main function of WAT is to store energy, while BAT can dissipate energy as heat through mitochondrial uncoupled respiration (5, 6). In recent years, beige adipocytes have been described as a third type of adipose cell, which can be transformed from white adipocytes and have a thermogenic function (7–10). These inducible beige adipocytes share several biochemical features with BAT, such as the ability to dissipate energy through the uncoupling protein 1 (UCP-1)-mediated uncoupling of oxidative phosphorylation to maintain body temperature (11, 12).

Irisin, an exercise-driven hormone, was first identified in 2012 and was presumably cleaved from its precursor protein fibronectin type III domain containing 5 (FNDC5) (13). The main function of irisin is to induce the “browning” of white adipocytes by increasing UCP-1 and consequently increasing whole-body energy expenditure (14). Therefore, irisin has attracted much attention in the treatment of obesity and related metabolic diseases (15). In addition to its beneficial effect on obesity, irisin has also been linked to positive effects on many other diseases in which exercise is beneficial, including type 2 diabetes mellitus (T2DM) (16, 17), cardiovascular disease (CVD) (18), nonalcoholic fatty liver disease (NAFLD) (19), Alzheimer’s disease (20), and metabolic bone diseases (15).

Increasing evidence has suggested that FNDC5 may have more than one type of transcript (20–23). Lourenco et al. reported two peptides that have characteristics of full-length FNDC5 and are not part of the irisin sequence, unlike the original report describing irisin as a cleavage product derived from FNDC5 (23). Indeed, Albrecht et al. also demonstrated the diversity of FNDC5 transcript variants (22). The existence and possible physiological functions of these FNDC5 variants in rodents and humans remain controversial and need to be further studied. Recently, our team identified a new FNDC5 variant in rat INS-1 cell lines while exploring the overlapping effects of GLP-1 and FNDC5 in fighting obesity. This novel FNDC5 variant lacks the transmembrane domain (exon 5), which makes this protein secretable. Due to this characteristic, we named this secretable FNDC5 variant sFNDC5. The potential anti-obesity functions of sFNDC5 have been preliminarily proven in our previous studies (24). Considering that sFNDC5’s major distinction from irisin is that it lacks the transmembrane domain while the majority of irisin sequences are shared, a range of similar functions, such as browning and lipolysis, and even its biological functions compared with irisin, need to be further explored. To explore the function of sFNDC5, we first developed an *in vitro* expression system and purification procedure.

There are numerous standardized systems for heterologous protein expression. The most widely used expression hosts are *Escherichia coli*, insect cells infected with baculovirus, mammalian cells, molds, and yeasts (25–27). Bacterial expression systems, such as the *Escherichia coli* expression system, are readily available and have unparalleled fast growth kinetics, inexpensive media, and high-level expression when producing a recombinant protein, but this system lacks the ability to create posttranslational modifications (28). Among the many posttranslational modifications that occur during protein expression, glycosylation is often important, and various glycosylation patterns can significantly affect protein functions, such as stability, folding, and secretion (29). Importantly, studies have shown that the lack of glycosylation decreases the secretion of irisin and is also related to the instability of its precursor protein FNDC5 (30). For providing posttranslational modifications of recombinant heterologous proteins, mammalian cell lines possess significant strengths. However, lower growth rates and expensive nutrient requirements limit their use in large-scale production (31). The yeast expression system, with its capability of performing many eukaryotic posttranslational modifications, including glycosylation, phosphorylation, proteolytic processing, and disulfide bond formation, offers an excellent recombinant eukaryotic protein expression system (32, 33). Among all yeast species, the methylotrophic yeast *Pichia pastoris*, with its characteristic of simple manipulation and high yield, is a widely recognized efficient protein production tool (33).

Therefore, in this work, we chose the methylotrophic yeast *Pichia pastoris* as an efficient tool for the large-scale production of high purity recombinant secretable FNDC5 (r-sFNDC5). The biological activities of r-sFNDC5 in energy expenditure, browning, and lipolysis were further explored and compared with those of irisin in adipocytes.

## Materials and Methods

### Expression Plasmid Construction and Transformation of *P. pastoris*

The rat r-sFNDC5 cDNA (167 amino acids) was designed and synthesized and then cloned into the EcoRI/XbaI site of pPICZαA (Invitrogen, USA). The resulting pPICZαA-sFNDC5 plasmid was transformed into *Pichia pastoris* X-33 competent cells following the manufacturer’s instructions (Pichia Easycomp Transformation Kit, Invitrogen, USA).

### Large-Scale Fermentation and Time Course Expression Study

The transformed *P. pastoris* r-sFNDC5 competent cells were selected on YPD (1% yeast extract, 2% peptone, 2% dextrose, and 2% agar) plates containing 100 μg/ml zeocin. After incubation for 2 to 3 days at 30°C, a single zeocin-resistant colony was selected for protein expression. The selected colony was cultured in 5 ml YPD medium (1% yeast extract, 2% peptone, 2% dextrose, 100 μg/ml zeocin) overnight under shaking (200 rpm 30°C). Then, 5 mL of yeast culture was transferred into a flask containing 50 mL of YPD medium (1% yeast extract, 2% peptone, 2% dextrose, 100 μg/ml zeocin) and cultured under shaking for another 12 to 14 h. Scale-up expression was performed by transferring 50 ml yeast solution into 500 ml YPD medium and culturing overnight. After the A280 value reached 12 to 18, the cells were harvested by centrifugation (8000 rpm for 10 min) and resuspended in 100 ml buffered methanol-complex medium (BMMY) (1% yeast extract, 2% peptone, 100 mM potassium phosphate (pH 6.0), 1.34% yeast nitrogen broth, 0.4 mg/L biotin and 0.5% methanol). Subsequently, the cells were incubated at 30°C for 4 days under shaking (200 rpm), and 0.5% methanol was added to the medium every day. The supernatants (1 ml) were collected once daily for A280 detection. The remaining samples were detected by SDS–PAGE analysis.

### Purification of r-sFNDC5

The r-sFNDC5 secreted into the medium was purified to homogeneity by a Ni-NTA resin column exchange method. On Day 4, yeast cultures were centrifuged (15 min, 8000 rpm), and the induced supernatant was dialyzed against 2 L buffer A (500 mM NaCl, 10 mM Tris, pH = 7.5) at 4°C overnight. The resulting supernatant was collected and loaded onto a Ni-NTA resin column (#L00250-C, GenScript, China) and washed with wash buffer (500 mM NaCl, 10 mM Tris, pH = 7.5) to remove impurities. His-tagged sFNDC5 was eluted with elution buffer (500 mM NaCl, 10 mM Tris, pH = 7.5, and 250 mM imidazole) and collected in 1.5 ml tubes (1 ml per tube). The collected samples were measured at A280 and then analyzed by 12% SDS–PAGE. The protein concentration was estimated by a BCA protein assay kit (#P1101, Beyotime).

r-irisin was expressed and purified using the same method as r-sFNDC5.

### Glycosylation Assay

To confirm glycosylation of the r-sFNDC5 protein, we treated the protein with recombinant N-glycanase (#P0704 L, PNGase F, New England BioLabs) and analyzed it by SDS–PAGE.

### Differentiation of 3T3-L1 Preadipocytes Into Mature Adipocytes

Murine preadipocyte (3T3-L1) cells (Chinese Academy of Sciences Cell Bank, Shanghai, China) were cultured in basic medium (DMEM supplemented with 10% bovine calf serum and 1% penicillin streptomycin) at 37°C in a humidified atmosphere of 5% CO_2_. To induce differentiation into adipocytes, cells were cultured in adipogenic differentiation induction medium (basic medium supplemented with 0.5 mM isobutyl methylxanthine, 0.25 μM dexamethasone and 5 μg/μl insulin). Three days after induction, the cells were switched to maintenance medium (basic medium supplemented with 5 μg/μl insulin only) and cultured for another two days. Then, the medium was changed to basic medium and cultured for several days until approximately 90% 3T3-L1 cells were adipogenic differentiated. Fully differentiated adipocytes were treated with r-sFNDC5, r-irisin, or vehicle for the indicated times. To demonstrate the effect of r-sFNDC5 on adipogenesis, the cells were treated with or without r-sFNDC5 at different concentrations throughout the differentiation period. Adipogenic differentiation was confirmed by Oil Red O staining (14, 34).

### Cell Proliferation Assay

3T3-L1 cells were seeded into a 96-well plate at a density of 1×10^4^ cells/well and treated with various concentrations of r-sFNDC5 (0, 20, 50, and 100 nM) and cultured for different times (8, 24, 48, and 96 h). Cell viability was detected by using the CCK-8 assay according to the manufacturer’s instructions.

### SDS–PAGE and Western Blotting

Total protein lysates of 3T3 cells were separated by SDS–PAGE (10-15%). After electrophoresis, proteins were transferred to PVDF membranes and incubated with primary antibodies at 4°C overnight. The membranes were incubated with an HRP-conjugated secondary antibody for another 1 h at room temperature. The antibodies were diluted to their appropriate ratio according to the manufacturer’s instructions. The bands were visualized with enhanced chemiluminescence substrate (Millipore). The antibodies used were as follows: FNDC5 (#ab174833, Abcam), UCP-1 (#U6382, Sigma), HSL (#ab109400, Abcam), perilipin (#ab3526, Abcam), adipoq (#ab22554, Abcam), and β-actin (#A5316, Sigma).

### RNA Isolation and RT–qPCR

Total RNA was isolated by TRIzol reagent (#15596018, Invitrogen) according to the manufacturer’s instructions. First-strand cDNAs were synthesized from 2 μg of total RNA using a High-Capacity cDNA Reverse Transcription Kit (#K1691, Invitrogen). RT–qPCR in triplicate was carried out with SYBR Green Master Mix (#A46113, Invitrogen). The 2^–△△Ct^ method was used to quantify the relative expression of the genes. β-actin was used as an internal control. The primers are listed in **Supplementary Table S1**.

### Immunofluorescence (IF) Staining of UCP-1

Differentiated mature 3T3-L1 adipocytes were treated with or without r-sFNDC5 for the indicated times. After fixation, the cells were incubated with UCP-1 antibody (1:200) overnight at 4°C. After washing, FITC-conjugated goat anti-rabbit IgG secondary antibody was incubated with the cells for another 1 h at RT. 4,6-Diamidino-2-phenylindole (DAPI) was used to counterstain the nuclei. Images were taken with a confocal laser microscopy system.

### Intracellular ATP Detection

ATP was measured in the cell lysates with an enhanced ATP assay kit (Beyotime) according to the manufacturer’s protocol.

### Statistical Analysis

All data are presented as the means ± SEM of at least three independent experiments. The statistical significance was analyzed by using GraphPad Prism 7.0 software, and comparisons between two groups were performed using one-way ANOVA followed by unpaired Student’s t-test. P < 0.05 was considered statistically significant.

## Results

### Sequence Analysis of sFNDC5

**Figure 1:**
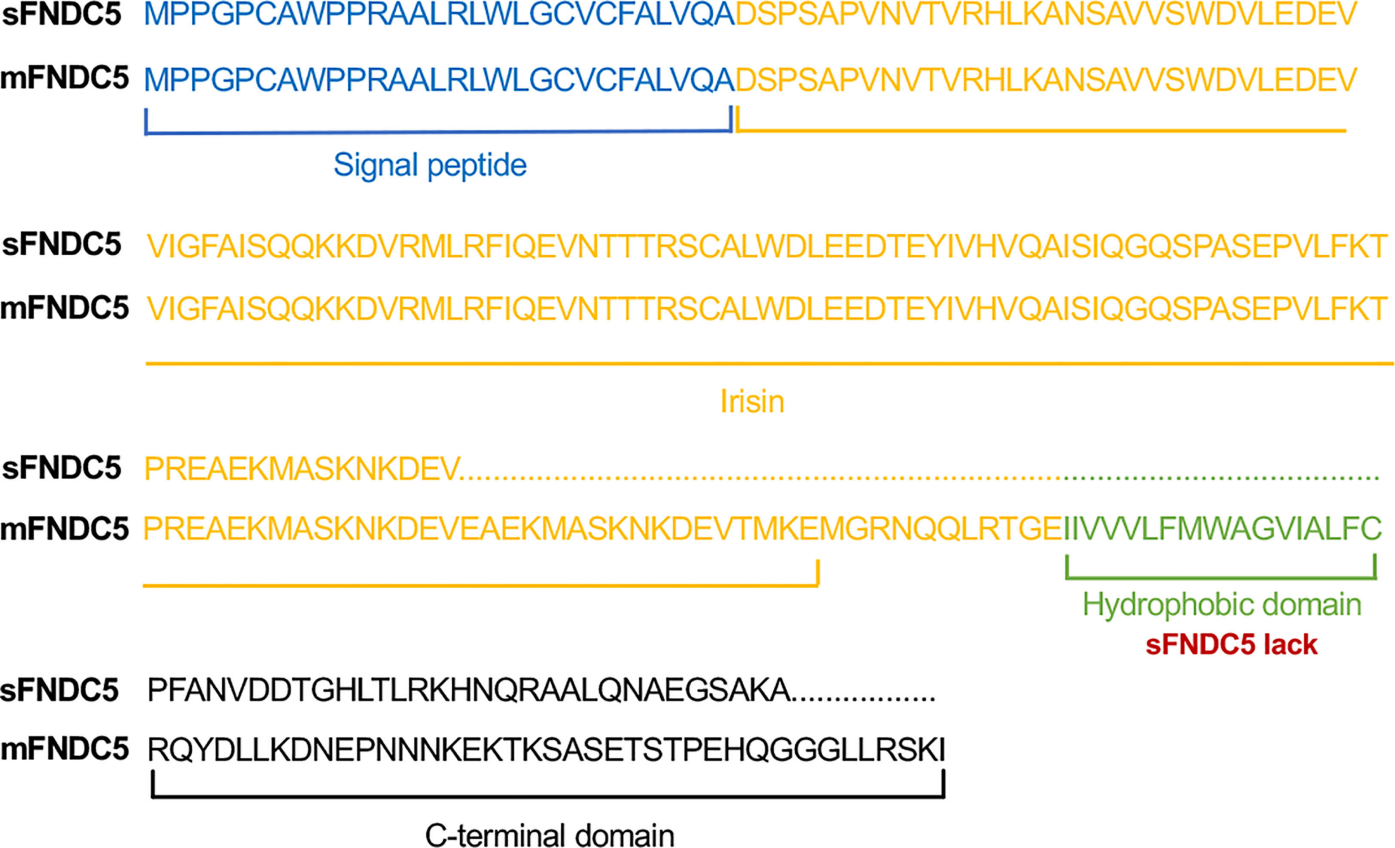
Schematic sequence of rat *FNDC5* variants. Schematic representation of the amino acid sequence alignment of sFNDC5 and mFNDC5. FNDC5 amino acid sequence with corresponding domains colored. Blue, signal peptide; Yellow, irisin; Green, hydrophobic domain; Black, C-terminal domain.

As described in our previous paper, sFNDC5 is derived from FNDC5 pre-mRNA through alternative splicing. Through alignment of the amino acid sequences of this new sFNDC5 transcript and membrane-bound FNDC5 (mFNDC5), we found that this variant lacks a transmembrane domain (exon 5) but shares most of the irisin sequence (**Figure 1**).

### Expression and Purification of r-sFNDC5 in *P. pastoris*

**Figure 2:**
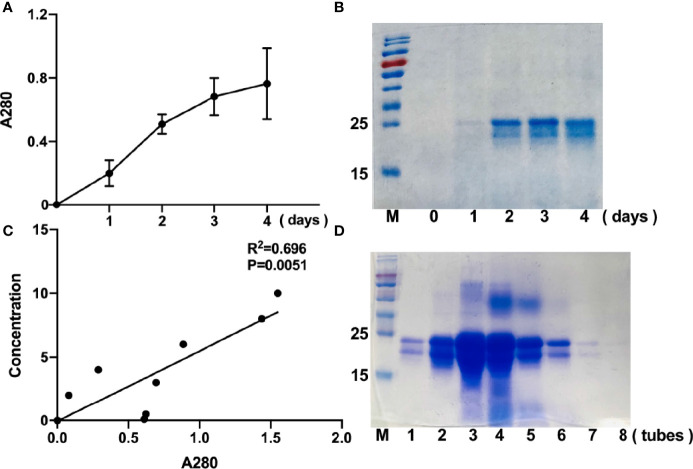
Time-course expression and purification of r-sFNDC5 in *P. pastoris.* Culture supernatants of r-sFNDC5 (1 ml) were collected from Day 1 to Day 4. **(A)** The supernatants were measured at A280 for relative quantification of the protein in the supernatants. **(B)** The proteins in the supernatants were analyzed by 15% SDS–PAGE and stained with Coomassie blue R250. Lanes 0 to 4 are representative supernatants after induction by methanol. **(C)** Elution curves of r-sFNDC5 from Ni-NTA resin in elution buffer. The eluents were collected and measured at A280 until the value of A280 was not increased, for a total collection of 8 ml. The eluent protein concentrations were also estimated by the BCA method and then analyzed by 15% SDS–PAGE. **(D)** 15% SDS–PAGE stained with Coomassie blue R250. Lanes 1-8, samples of purified r-sFNDC5 collected in sequence.

To achieve high-yield expression of r-sFNDC5 in a yeast expression system, the pPICZαA-sFNDC5 plasmid was designed and constructed. The yeast culturing and induction of protein expression were performed as described in the *Materials and Methods.* To determine the optimal time for the expression of r-sFNDC5, methanol-treated supernatant samples were collected on Day 1, Day 2, Day 3, and Day 4. By measuring A280, we found that the expression of r-sFNDC5 was time-dependent (**Figure 2A**). SDS–PAGE analysis showed purified r-sFNDC5 with a molecular weight range from 18 to 27 kDa (**Figure 2B**).

After 4 days of methanol induction, the culture medium was centrifuged, and the induced supernatants were collected and subjected to an Ni-NTA resin column for purification. The purified r-sFNDC5 was eluted from the column (1 ml per tube) and measured for A280 absorbance readings, and the BCA protein assay was used for quantification. The A280 absorbance readings correlated well with the protein concentration, which may be used as a rapid method to determine the elution concentration (**Figure 2C**). The samples collected during the elution peak were selected for analysis by 12% SDS–PAGE and stained with Coomassie brilliant blue (**Figure 2D**).

### N-Linked Glycosylation Analysis of r-sFNDC5

**Figure 3:**
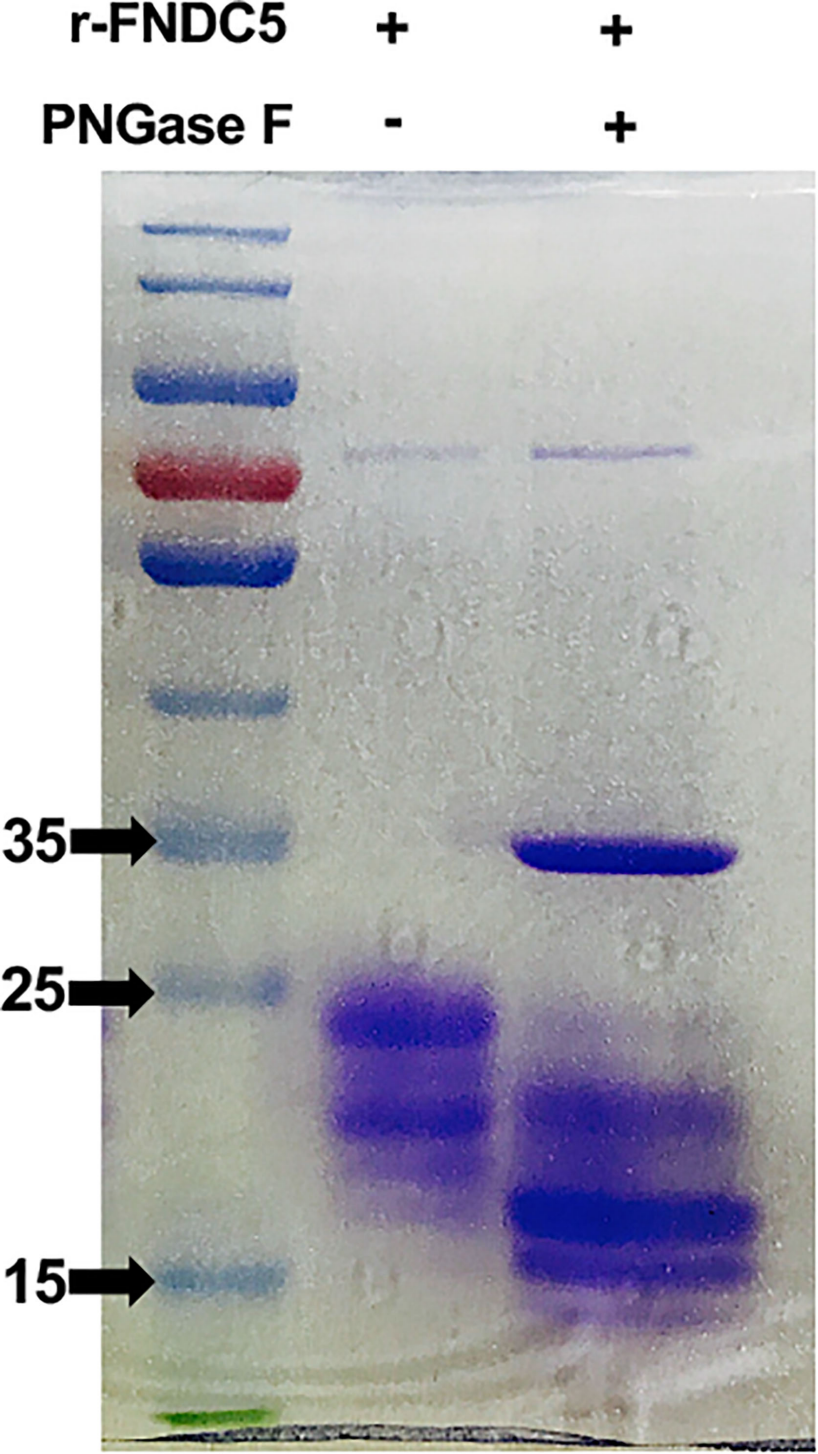
N-linked glycosylation of the r-sFNDC5. Purified r-sFNDC5 protein was incubated with or without PNGase F at 37°C for 1 h, and glycosylation was confirmed by Coomassie blue staining.

To examine whether the higher bands of r-sFNDC5 were glycosylated, purified r-sFNDC5 was treated with or without the enzyme N-glycosidase F (PNGase F) for 1 h and subjected to SDS–PAGE analysis. The enzyme-treated r-sFNDC5 exhibited a single band with the expected molecular mass of 18 kDa (**Figure 3**), confirming that the 20-27 kDa mass of r-sFNDC5 expressed by *P. pastoris* was mainly the result of N-glycosylation.

### r-sFNDC5 Stimulates Browning and Lipolysis in Differentiated Mature 3T3-L1 Cells

**Figure 4:**
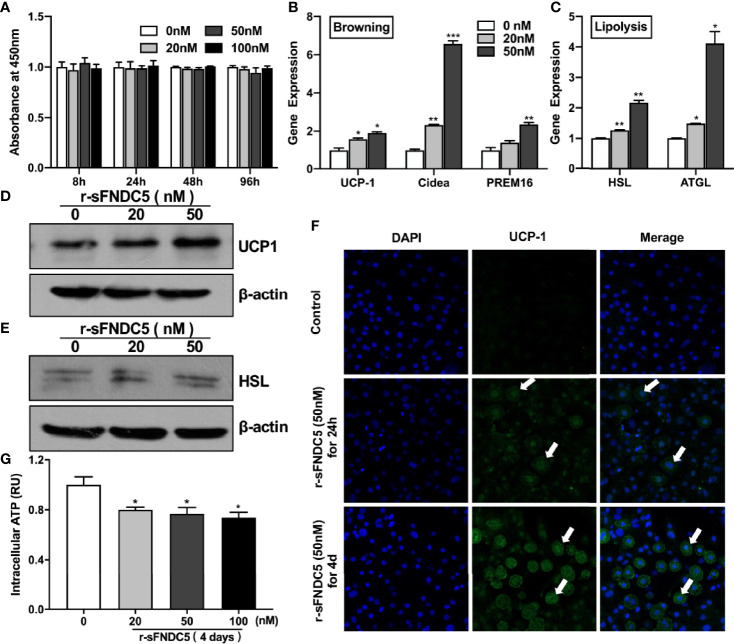
r-sFNDC5 induced the browning and lipolysis of 3T3-L1 adipocytes. The biological activity of purified r-sFNDC5 was assessed in differentiated 3T3-L1 adipocytes. **(A)** 3T3-L1 preadipocytes were treated with various concentrations of r-sFNDC5 for the indicated times, and cell viability was assessed using a CCK-8 assay. The data are expressed as OD values at 450 nm. After fully differentiating, mature 3T3-L1 adipocytes were treated with r-sFNDC5 (20 nM and 50 nM) for 8 h. Then, the relative mRNA levels of browning genes **(B)** and lipolysis genes **(C)** were measured by RT–qPCR, and **(D, E)** western blotting was performed for UCP-1 and HSL. β-actin expression was used as a control. **(F)** Representative 3T3-L1 adipocytes immunostained for UCP-1 (green) and nuclei (blue) after r-sFNDC5 (50 nM) treatment for 24 h or 4 days. White arrows indicate UCP-1-positive cells. Images were taken using a confocal fluorescence microscope. **(G)** ATP levels measured in lysates of 3T3-L1 adipocytes treated with 20-100 nM r-sFNDC5 for 4 days. ATP concentrations were normalized to protein content and control. Each experiment was repeated three times. Values are the mean ± SEM. *P < 0.05, **P < 0.01 and ***P < 0.001 vs. control.

To verify the protein activity and functions of our purified r-sFNDC5, we first evaluated the influence of r-sFNDC5 on cell viability. 3T3-L1 cells were treated with different concentrations of r-sFNDC5 for the indicated times, and cell viability was assessed using the CCK-8 assay. The results showed no effect on cell viability at these concentrations of r-sFNDC5 treatment, which indicated no toxicity of this protein (**Figure 4A**). Therefore, we used concentrations of 20 and 50 nM in subsequent studies. We found that r-sFNDC5 induced a rapid upregulation of browning (UCP-1, PRDM16, Cidea) and lipolysis-related genes (ATGL, HSL) in differentiated mature 3T3-L1 adipocytes after treatment for 8 h (**Figures 4B, C**). Consistent with changes in their transcription levels, UCP-1 and ATGL protein levels were also significantly enhanced (**Figures 4D, E**). Immunofluorescence staining of UCP-1 further confirmed a significantly higher level of expression after 24 h of treatment with r-sFNDC5 (**Figure 4F**, middle panel), and its level dramatically increased after 4 days of r-sFNDC5 treatment (**Figure 4F**, bottom panel).

To further characterize the impact of r-sFNDC5 on cellular energy metabolism, we subsequently measured intracellular ATP. The results showed that intracellular ATP levels were decreased with r-sFNDC5 treatment (**Figure 4G**). The reason is that sFNDC5 induces fast substrate oxidation with a low rate of ATP production due to increased UCP1 expression (35).

### r-sFNDC5 Inhibits Adipogenic Differentiation of 3T3-L1 Cells

**Figure 5:**
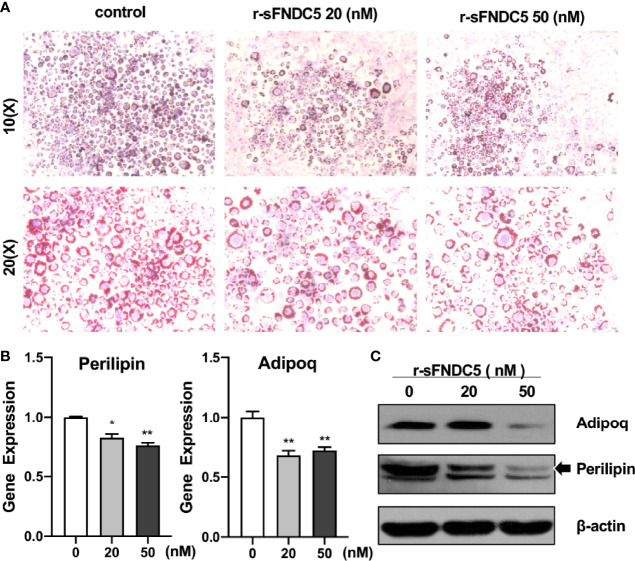
r-sFNDC5 suppresses the differentiation of 3T3-L1–derived adipocytes. Human visceral preadipocytes were induced to adipogenic differentiation with or without irisin (50 nM) for 18 days. r-sFNDC5 (20 nM or 50 nM) was added to 3T3-L1 cells with adipogenesis induction medium. **(A)** Cells were stained with Oil Red O to visualize lipid droplets. **(B, C)** Relative mRNA and protein levels of the perilipin and adiponectin genes were measured by RT–qPCR and western blotting. β-actin expression was used as a control. Each experiment was repeated three times. Values are the mean ± SEM. *P < 0.05 and **P < 0.01 vs. control.

In addition to exploring the function of r-sFNDC5 on differentiated mature adipocytes, the effect of r-sFNDC5 on lipid accumulation during adipogenic differentiation was further studied. The 3T3-L1 cells were treated with different concentrations (20 nM and 50 nM) of r-sFNDC5 throughout the differentiation period. As shown in **Figure 5A**, adipocyte accumulation was reduced in the presence of r-sFNDC5 after 10 days of differentiation. Moreover, the expression of Perilipin and Adipoq was also reduced at both the gene and protein levels (**Figures 5B, C**). The former coats the surface of intracellular lipid droplets, and the latter is a key gene related to lipid metabolism and adipogenesis. Collectively, our results demonstrated that r-sFNDC5 exerts an inhibitory effect on preadipocyte adipogenic differentiation.

### Functional Comparison of r-sFNDC5 and r-Irisin in Differentiated Mature 3T3-L1 Adipocytes

**Figure 6:**
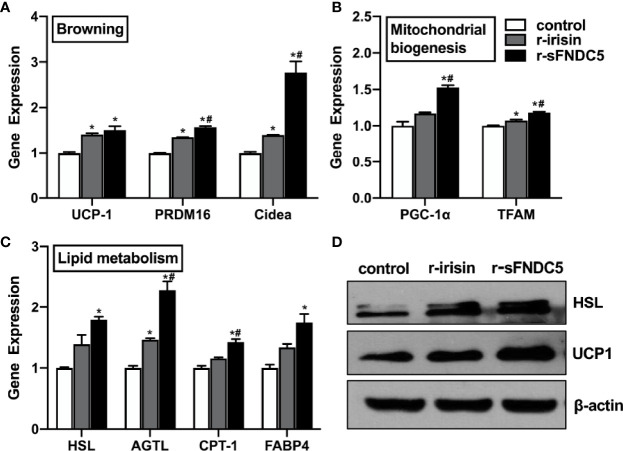
The function comparison of r-sFNDC5 and r-irisin in the 3T3-L1 adipocytes. After fully differentiated, 3T3-L1 adipocytes were treated with r-sFNDC5 (20 nM) or r-irisin (20 nM) for 8 h. **(A)** Relative mRNA levels of browning genes, **(B)** mitochondrial biogenesis, and **(C)** lipid metabolism were measured by RT–qPCR. **(D)** The contents of UCP-1 and HSL were measured using western blotting. β-actin expression was used as a control. The asterisk (*) above the bar denotes statistically significant differences in mRNA levels calculated relative to the control, while the hash (#) denotes statistically significant differences calculated between the irisin and sFNDC5 groups. Each experiment was repeated three times. Values are the mean ± SEM. *P < 0.05 vs. control, ^#^P < 0.05 vs. irisin.

Next, we sought to compare the biological function of r-sFNDC5 with r-irisin, as the browning and lipolysis functions of r-sFNDC5 had been verified above. We treated differentiated mature 3T3-L1 adipocytes with 20 nM r-sFNDC5 and r-irisin for 8 h and found that both proteins induced the expression of genes related to browning (*UCP1, Cidea, PRDM16*), mitochondrial biogenesis (*PGC1α, TFAM*), and lipid metabolism (*ATGL, HSL, CPT-1, FABP4*) (**Figures 6A**–**C**). However, r-sFNDC5 had a much stronger effect than r-irisin. Western blot results showed that the levels of HSL and UCP1 were also increased more significantly after r-sFNDC5 treatment (**Figure 6D**). Overall, these results suggested that r-sFNDC5 exhibited superior biological activity to r-irisin.

## Discussion

With the discovery of irisin, researchers have reported that irisin plays a pivotal role in fat browning and the regulation of energy expenditure and has the potential to be used as a promising therapeutic agent in the treatment of metabolic and endocrine disorders. However, there is still considerable heterogeneity in reports on the molecular weights of different forms of irisin and its precursor FNDC5 in humans and mice (20, 22, 36). Many studies have detected FNDC5 in a molecular weight range from 22 to 30 kDa in untreated muscle of different species (37–41). Moreover, one study detected irisin with a molecular weight up to 75 kDa in hippocampal cells of mice using a commercial anti-FNDC5 antibody. Through analysis by MS, they speculated that FNDC5 in the brain appears to exist as an uncut transmembrane protein, as 2 peptides that are characteristic of full-length FNDC5 were found in all western blotting bands (20). The lack of reliable antibodies for the detection of irisin is a major reason for the discrepancies (42). Other explanations for the inconsistent MW results may be due to site-directed mutation, irisin dimer, and glycosylated irisin. As the proteolytic enzyme that cleaves irisin from FNDC5 has yet to be identified and the *fndc5* gene is known to produce diverse transcripts, there may exist other soluble uncut FNDC5 isoforms in addition to proteolysis. Indeed, Albrecht et al. reported that there is a greater transcript diversity of human FNDC5 than currently annotated (22). They reported that some aberrant transcripts were changed only in the C-terminal region and did not affect the irisin sequence, and some lacked the signal peptide and had truncated irisin (21). However, all of this is speculation, and the existence and possible physiological functions of various FNDC5 transcripts in rodents and humans have been a matter of controversy.

In our previous study, a new FNDC5 transcript from rat INS-1 cell lines was identified by RT–qPCR analysis. According to the contrast in the schematic sequence between irisin and sFNDC5, this new variant shares most of the irisin sequence (24). To clarify the precise biological function of this transcript, we first obtained the sFNDC5 protein with a *P. pastoris* yeast expression system and purified it with a Ni-NTA column, which is a useful experimental tool for heterogeneous protein production (43). Therefore, in this paper, we introduced this protein expression and purification procedure in detail. We successfully used this expression system to produce a high yield of r-sFNDC5, providing the ability to explore its biological function in subsequent studies. R-sFNDC5 is a glycoprotein, as SDS–PAGE analysis showed a molecular weight range from 18 to 27 kDa. Unfortunately, we could not distinguish sFNDC5 from other FNDC5 derivatives due to a lack of specific antibodies. Treatment of r-sFNDC5 with PNGase F caused a decrease in the molecular mass to approximately 18 kDa, confirming that r-sFNDC5 is a glycosylated protein. The main band in **Figure 3** supports the existence of deglycosylated sFNDC5 at a size of 15-16 kDa. There is still a shallow band at 20 kDa, probably because PNGase F is an enzyme that removes N-linked oligosaccharides but not other oligosaccharides; this band is therefore the result of incomplete deglycosylation (44). In addition, we found that the concentration of r-sFNDC5 in each elution collection tube was positively correlated with the relative change in the measured A280 absorbance readings. This not only provides a rough estimate of eluted protein concentration based on the absorbance of A280 but also ensures that the protein is completely eluted. This method is simple and can be done quickly.

Despite numerous studies on FNDC5 variants (21, 45), the biological function of these forms is still poorly understood. Studies have confirmed that irisin can promote white adipose tissue browning (13, 46), stimulate lipolysis (47–49), and thus play a critical role in regulating energy homeostasis (50). Therefore, we examined the biological activity of r-sFNDC5 on browning and lipid metabolism in 3T3-L1 adipocytes. From the results, we found that r-sFNDC5 activated the expression of browning (UCP-1, PREM16, Cidea)- and lipolysis (HSL and ATGL)-related genes and proteins. UCP1 is a protein that is essential for brown fat cells and is localized to the mitochondrial inner membrane, where it uncouples cellular respiration and mitochondrial ATP synthesis to dissipate heat instead of generating ATP (35). Indeed, depletion of intracellular ATP levels was also found in r-sFNDC5-treated cells, which further indicated that r-sFNDC5 treatment induced high expression levels of UCP-1. In our study, we also explored the effect of r-sFNDC5 on preadipocyte adipogenic differentiation. As expected, lipid droplets dramatically increased during 3T3-L1 preadipocyte adipogenic differentiation, accompanied by upregulated expression of adipose-related genes, whereas the levels showed a downward trend after r-sFNDC5 treatment, suggesting that r-sFNDC5 inhibits preadipocyte adipogenic differentiation.

Irisin and sFNDC5 are both FNDC5 derivatives, and thus it is necessary to compare the effects of these two forms of derivatives on metabolism-related functions. As our previous study proved that irisin at 20 nM effectively upregulated UCP-1 expression, we compared its biological function with r-sFNDC5 at this concentration (14). As expected, r-irisin increased browning, lipolysis, and mitochondrial biogenesis genes at both the transcriptional and protein levels. However, r-sFNDC5 had a much stronger effect than irisin in this respect. Due to the lack of a transmembrane region, sFNDC5 can be readily secretable without cleavage, which may affect its biological functions.

In summary, we have described a highly efficient production and purification system for the preparation of r-sFNDC5. Its biological activities were further confirmed not only in mature adipocytes but also in preadipocytes undergoing adipogenic differentiation. Additionally, r-sFNDC5 was proven superior to r-irisin in terms of functions related to lipid metabolism. Clarifying whether sFNDC5 plays a significant and beneficial role in other tissues, the existence and functions of this secreted FNDC5 protein in humans and mice, and the specific mechanism of sFNDC5 underlying metabolism-related effects requires further research. The present findings provide preliminary experimental evidence for the potential use of this secreted FNDC5 derivative (sFNDC5) for the treatment of obesity and obesity-related metabolic disorders.

## Data Availability Statement

The original contributions presented in the study are included in the article/supplementary material. Further inquiries can be directed to the corresponding author.

## Author Contributions

HL, SL, and DT contributed to conceptualization, methodology, supervision, validation, and manuscript development. YZ contributed to research and investigation including most cell culture experiments, formal analysis of results, data curation, and writing the manuscript. WD contributed to revising the manuscript. All authors read and approved the final manuscript. DT is the guarantor of this work and has full access to all the data in the study and takes responsibility for the integrity of the data and the accuracy of the data analysis.

## Funding

The work was supported by National Natural Science Foundation of China Grants (81970743).

## Conflict of Interest

The authors declare that the research was conducted in the absence of any commercial or financial relationships that could be construed as a potential conflict of interest.

## Publisher’s Note

All claims expressed in this article are solely those of the authors and do not necessarily represent those of their affiliated organizations, or those of the publisher, the editors and the reviewers. Any product that may be evaluated in this article, or claim that may be made by its manufacturer, is not guaranteed or endorsed by the publisher.
