# Supplementary material for: Expression of Recombinant Rat Secretable FNDC5 in Pichia Pastoris and Detection of Its Biological Activity
Source: Front Endocrinol (Lausanne). 2022 Mar 7;13:852015. doi: 10.3389/fendo.2022.852015 (PMC8936140; doi:10.3389/fendo.2022.852015)
Supplement: Supplementary file 1 [file Table_1.docx]

| Gene | Primer sequence |
| --- | --- |
| UCP-1 F | CGTCCCCTGCCATTTACTGT |
| UCP-1 R | GACCCGAGTCGCAGAAAAGA |
| Cidea F | TCACAACTGGCCTGGTTACG |
| Cidea R | CCTTGAAGCTTGTGCATCGG |
| PRDM16 F | CCCCACATTCCGCTGTGAT |
| PRDM16 R | CTCGCAATCCTTGCACTCA |
| HSL-F | GTGGCGAAAAGGCAAGATCA |
| HSL-R | TTCCCGAACACCTGCAAAGA |
| ATGL-F | GACAGCTCCACCAACATCCA |
| ATGL-R | GCAAAGGGTTGGGTTGGTTC |
| FABP4-F | TCACCTGGAAGACAGCTCCT |
| FABP-4-R | AATCCCCATTTACGCTGATG |
| CPT-1-F | TGTCCATCATGGCTTGTCTC |
| CPT-1-R | AGTACCCAAGCGTACCAAGC |
| PGC-1α F | CCCTGCCATTGTTAAGACC |
| PGC-1α R | TGCTGCTGTTCCTGTTTTC |
| Adipoq F | GCACTGGCAAGTTCTACTGCAA |
| Adipoq R | GTAGGTGAAGAGAACGGCCTTGT |
| Perilipin F | GGATGGAGACCTCCCTGAG |
| Perilipin R | CTCACAGGTCCCGCTCAC |
| TFAM F | ATTAGGAGGGTCTCGCTCCA |
| TFAM R | GGCCATGCAAGGCTTTTCC |
| β-actin F | GACCTCTATGCCAACACAGT |
| β-actin R | AGTACTTGCGCTCAGGAGGA |

**Table S1. Quantitative real-time PCR gene-specific primer sequences.**
